# Printability Metrics and Engineering Response of HDPE/Si_3_N_4_ Nanocomposites in MEX Additive Manufacturing

**DOI:** 10.3390/nano14201680

**Published:** 2024-10-19

**Authors:** Vassilis M. Papadakis, Markos Petousis, Nikolaos Michailidis, Maria Spyridaki, Ioannis Valsamos, Apostolos Argyros, Katerina Gkagkanatsiou, Amalia Moutsopoulou, Nectarios Vidakis

**Affiliations:** 1 Department of Industrial Design and Production Engineering, University of West Attica, 122 43 Athens, Greece; v.papadakis@uniwa.gr; 2 Foundation for Research and Technology Hellas (FORTH), Institute of Electronic Structure and Laser (IESL), 70013 Heraklion, Greece; 3 Department of Mechanical Engineering, Hellenic Mediterranean University, 71410 Heraklion, Greece; markospetousis@hmu.gr (M.P.); mspyridaki@hmu.gr (M.S.); valsamos@hmu.gr (I.V.); gkagka@hmu.gr (K.G.); amalia@hmu.gr (A.M.); 4 Physical Metallurgy Laboratory, Mechanical Engineering Department, School of Engineering, Aristotle University of Thessaloniki, 54124 Thessaloniki, Greece; nmichail@auth.gr (N.M.); aargyros@auth.gr (A.A.); 5 Centre for Research & Development of Advanced Materials (CERDAM), Center for Interdisciplinary Research and Innovation, Balkan Centre, Building B’, 10th km Thessaloniki-Thermi Road, 57001 Thessaloniki, Greece

**Keywords:** high-density polyethylene (HDPE), silicon nitride (Si_3_N_4_), material extrusion (MEX), three-dimensional printing (3D-P), structural characterization, morphological analysis

## Abstract

Herein, silicon nitride (Si_3_N_4_) was the selected additive to be examined for its reinforcing properties on high-density polyethylene (HDPE) by exploiting techniques of the popular material extrusion (MEX) 3D printing method. Six different HDPE/Si_3_N_4_ composites with filler percentages ranging between 0.0–10.0 wt. %, having a 2.0 step, were produced initially in compounds, then in filaments, and later in the form of specimens, to be examined by a series of tests. Thermal, rheological, mechanical, structural, and morphological analyses were also performed. For comprehensive mechanical characterization, tensile, flexural, microhardness (M-H), and Charpy impacts were included. Scanning electron microscopy (SME) was used for morphological assessments and microcomputed tomography (μ-CT). Raman spectroscopy was conducted, and the elemental composition was assessed using energy-dispersive spectroscopy (EDS). The HDPE/Si_3_N_4_ composite with 6.0 wt. % was the one with an enhancing performance higher than the rest of the composites, in the majority of the mechanical metrics (more than 20% in the tensile and flexural experiment), showing a strong potential for Si_3_N_4_ as a reinforcement additive in 3D printing. This method can be easily industrialized by further exploiting the MEX 3D printing method.

## 1. Introduction

Additive manufacturing (AM) [1] can provide the scientific community with multiple customized products and complex constructs with polymers that have advanced properties, or composites [2,3,4,5] that are suitable for various applications [6]. It is also called three-dimensional printing (3D-P) and is currently a manufacturing technology based on data derived from digital three-dimensional geometric models. These are exploited to manufacture objects layer-by-layer [7].

Some of the AM application fields are automotive, aerospace, marine [8], and medical [9]. There has also been significant progress in the 3D printing of ceramics [10,11], metals (such as aluminum alloys) [12,13], and polymeric materials (such as acrylonitrile butadiene styrene—ABS—or polymeric blends) [14,15]. Polymers are considered to be a wide group of materials that are composed of many smaller molecules, known as monomers, to create long chains [16]. The polymer characteristics can be improved by introducing ceramic particles and producing their respective polymeric composites [17].

Existing natural polymers, as well as synthetic ones, are useful in applications related to medicine, communication, nutrition, clothing, containers, transportation, and buildings [16]. The most well-known thermoplastic polymers used as matrix materials are acrylonitrile butadiene styrene (ABS) [18], polylactide (PLA) [18], polyetherimide (PEI), polycarbonate (PC) [19], high-density polyethylene (HDPE) [20], polyamide, polypropylene (PP) [21], and polycaprolactone (PCL) [22].

HDPE is a hard semicrystalline thermoplastic categorized as a thermoplastic. It can be utilized to produce various parts, such as bucket pipes, rigid bottles [23], or even human implants [24]. The application amplitude it covers includes objects of packaging industries, jugs, chemical containers, household utilities, and engineering structural applications (with materials such as sandwich composites or lightweight composite foams) [25,26]. HDPE possesses useful properties, such as low density, high chemical resistance, electrical insulation, ease of moldability [27], water resistance, and thermal stability [28,29]. HDPE was employed as the polymeric matrix for the development of compounds with MEX technology; with fillers, such as the eco-friendly and sustainable biochar [30]; copper [31], to induce multi-functionality; or ceramic fillers such as titanium dioxide [32] and titanium nitride [33]. In all research, HDPE has proven to be a valuable matrix material, and the composites improved its performance, making it more efficient in the applications it is suitable for.

Silicon nitride (Si_3_N_4_) is a bioceramic material suitable for several potential applications [7], particularly in the biomedical field, where it has been examined for several years (for example, against gram-negative Escherichia Coli, in dental implants, osteofixation systems, osteogenesis, in vitro studies, or orthopedic applications) [34,35,36,37,38,39,40,41,42,43,44]. Its high strength, osteoconductivity, and antibacterial characteristics are useful for its utilization in applications such as spinal implants [42,45,46]. Moreover, it has been proven over multiple years that the properties of silicon nitride make it suitable for use in bone tissue, and it promotes osteogenic activity [47,48]. It exhibits excellent mechanical performance, microstructural characteristics, and biological activity [39,41]. Because of its excellent mechanical and tribological properties, it is considered a very promising reinforcement material for the fabrication of polymer/ceramic filaments [17,49,50,51].

By searching the bibliography, HDPE/Si_3_N_4_ has been developed with different methods, and not for AM [52,53]. In MEX 3D printing [54] silicon nitride ceramic nanoparticles were combined with high-density polyethylene or polypropylene to create composite filaments of 5 and 10 vol. %. According to the results, the wear tests of the polyethylene/Si_3_N_4_ composite proved the positive influence of the increasing ceramic content of the filament on the wear resistance. Apart from the wear tests, mechanical tests, such as tensile experiments, were carried out in the filament only, while case studies’ parts were fabricated for the proof of concept, with various (non-standard) geometries. Additionally, it has been examined as a mechanical properties enhancement filler in AM on various thermoplastics. These include polyethylene terephthalate glycol (PETG) [55], PLA [56], polypropylene (PP) [57], and ABS [58] in the MEX method or biomedical resins in the AM method of vat photopolymerization (VPP) [59].

The bibliography review revealed several studies for HDPE and Si_3_N_4_ in AM, but still, no research refers to HDPE/Si_3_N_4_ nanocompounds for the 3D printing of MEX, with an evaluation of the mechanical response of the 3D-printed examples, their quality characteristics, and full characterization of the nanocomposites. Therefore, this study examined the influence of silicon nitride on the behavior and characteristics of HDPE, employing MEX 3D printing techniques for the preparation of the respective composites. Six different filler percentages were utilized between 0.0 wt. % and 10.0 wt. % (step 2.0). The HDPE and Si_3_N_4_ raw materials were prepared and mixed, to be extruded into the respective filaments. The next step included the utilization of the filament to support the specimen fabrication procedure. Subsequently, the specimens were subjected to testing for multiple characteristics related to their mechanical behavior and morphological and structural characteristics. Additionally, samples from all of the created composites underwent an examination of their chemical composition through energy-dispersive spectroscopy (EDS), their rheological properties through viscosity and material flow rate (MFR) analysis, and their thermal response by implementing measurements through the method of thermogravimetric analysis (TGA) and the method of differential scanning calorimetry (DSC).

The investigated mechanical characteristics included Young’s and flexural modulus, ultimate tensile strength (UTS), flexural and Charpy impact strength, tensile toughness, and microhardness (M-H). The morphological investigation was conducted using scanning electron microscopy (SEM), which produced images of multiple magnifications, revealing the lateral and fracture sections of the specimens. The structural characteristics were examined by evaluating the porosity (voids in the 3D printing structure) and geometrical accuracy levels of the examples after microcomputed tomography (μ-CT). These metrics pertain to the quality of the manufactured components. The main objectives of the study were to

- Evaluate the reinforcement effectiveness of Si3N4 on the HDPE polymer on nanocomposites prepared with MEX methods for the 3D printing technology.
- Characterize the nanocomposites produced within the context of the research.
- Assess the impact of Si3N4 on quality metrics, such as the geometric deviation and the voids (porosity) of the 3D-printed items.
- Produce HDPE/Si3N4 nanocomposites with improved characteristics, to enhance the robustness of HDPE in respective applications, employing the MEX technique in 3D printing.

## 2. Materials and Methods

**Figure 1:**
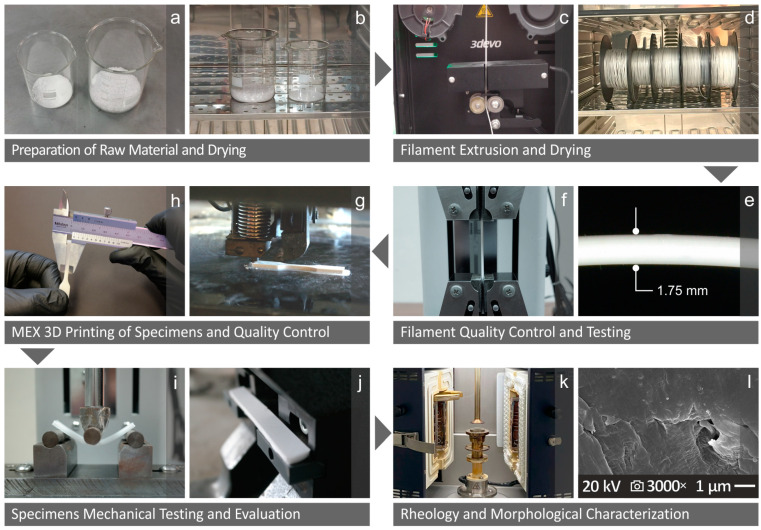
Introduction to the main procedures conducted herein, as follows: (**a**) preparing and (**b**) drying of the unprocessed HDPE and Si_3_N_4_, (**c**) filament fabrication, (**d**) drying process, (**e**) filaments’ quality inspection, and (**f**) testing; (**g**) specimens’ fabrication with 3D-P and (**h**) examples quality inspection; (**i**–**l**) specimens’ mechanical experiments and assessment, (**k**) rheology, and (**l**) morphology inspection.

The series of procedures and tests implemented in this work are presented in Figure 1. The preparation procedure and drying of HDPE and Si_3_N_4_ are illustrated in Figure 1a and Figure 1b, respectively. The extrusion, drying, quality control, and testing of the corresponding filaments are presented in Figure 1c–f. The MEX (material extrusion) 3D fabrication of the specimens is depicted in Figure 1g, along with their quality inspection in Figure 1h. The mechanical properties examination and evaluation of the specimens are shown in Figure 1i,j, while the characterization of rheology and morphology are presented in Figure 1k,l.

### 2.1. Materials

The polymer employed as the matrix material in the study, i.e., high-density polyethylene polymer (brand name Kritilen), was supplied by the company Plastika Kritis S.A., located in Heraklion, Crete, Greece. It was in the form of powder, and industrial grade, and its characteristics, based on the available data from the supplier, were a mass-flow rate of 7.5 g/10 min, a density of 0.960 g/cm^3^, and a Vicat softening temperature of 127 °C. Its tensile strength is 29 MPa (ASTM D638 [60]), and its impact strength is 77 kJ/m^2^.

Silicon nitride (Si_3_N_4_) nanoparticles were supplied by Nanographi (Ankara, Turkey). Nanoparticles have a purity of 99.6% and a size of 760 nm, as confirmed using SEM and depicted below.

### 2.2. Preparation of HDPE/Si_3_N_4_ Filament and 3D Printing

The initial step was to prepare HDPE and Si_3_N_4_ unprocessed materials in proper quantities, and then proceed to mix them for approximately 20 min at 4000 rpm with a high-wattage blender. The filler quantities were selected based on tests that were conducted in advance, where samples of gradually increasing filler quantities were created and mechanically tested for their properties. The procedure ended by the time the mechanical properties of the tested samples did not show further improvement, as this would suggest that the filler saturates in the matrix, and thus further enhancement is expected [61,62].

Fabrication of the filaments followed the next procedure, which was supplied by the already prepared and dried mixtures of the raw materials. The unfilled HDPE and HDPE/Si_3_N_4_ 2.0, 4.0, 6.0, 8.0, and 10.0 wt. % filaments were extruded by an extrusion Noztek Pro system from Noztek, located in Shoreham-by-Sea, UK. All filaments were shredded into pellets (3devo shredder, Utrecht, The Netherlands) and dried at 80 °C before being supplied to the extruder 3D Evo Composer 450 from 3D Evo, located in B. V., NL, to produce the filament for the 3D printing of the test examples.

During filament extrusion, their diameters were monitored using a built-in sensor to detect any necessary micro-adjustments and change the extrusion speed. All of the filaments achieved a diameter of 1.65–1.85 mm, which is acceptable for the 3D fabricating procedure of the specimens. The extrusion parameters were provided by the information found in the existing literature [31,32,33,63]. Overall, the Si_3_N_4_ nanoparticles dispersion was ensured in the HDPE thermoplastic by a three-step process, which is as follows:

1. Mixing of unprocessed materials.
2. Thermomechanical mixing through the first extrusion process (Noztek extruder).
3. Thermomechanical mixing through the second extrusion process (3Ddevo extruder, which, additionally, is specially designed for materials mixing by itself, through the specially designed screw it uses).

To validate the uniformity of the nanoparticles in the matrix, the internal structure of the samples was inspected with SEM, as explained below. Finally, the deviation on the mechanical tests was validated, since high deviation in the experiments would indicate different compositions in the tested examples.

An Intamsys Funmat HT 3D printing machine functioning with the FFF technology, by Intamsys Technology Co. Ltd., located in Shanghai, China, was employed for the manufacturing of the 3D-P specimens. The selected 3D-P parameters followed the designed model of the specimens; the printed specimens are presented in the Supplementary Materials (Figure S1) of this work.

### 2.3. Morphological and Chemical Analysis

To observe the specimens, as well as the morphology of the Si_3_N_4_ raw material (nanoparticles), SEM images were captured. For the specimens, images were obtained from the fractures and their lateral sections. The device utilized was a JSM-IT700HR field emission SEM microscope by the JEOL company, located in Tokyo, Japan, which was also responsible for deriving the composition of the examined samples and the raw material of Si_3_N_4_ with EDS mapping. The working conditions during the two analyses were high-vacuum mode, 5 kV acceleration voltage, and gold sputtering of the samples.

**Figure 2:**
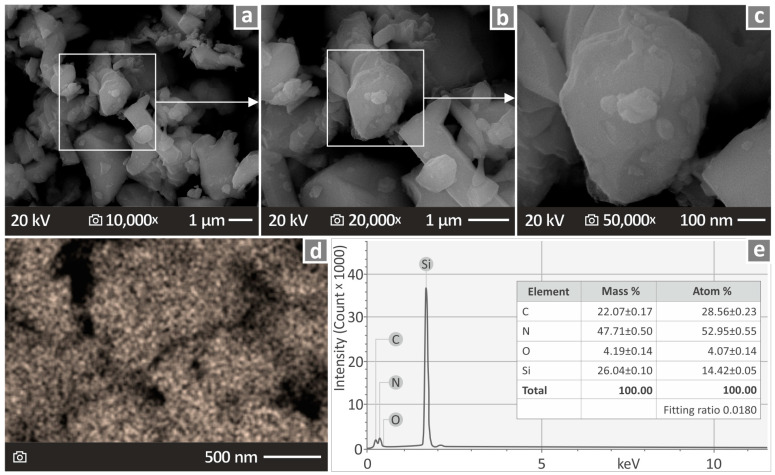
(**a**–**c**) SEM images of the Si_3_N_4_ nanoparticles in 10K×, 20K×, and 50K× magnifications, (**d**) an EDS mapping, and (**e**) chemical analysis of Si_3_N_4_.

Figure 2a–c show three SEM images of Si_3_N_4_ at 10K×, 20K×, and 50K× magnifications. Figure 2d presents an EDS mapping of the Si element, and Figure 2e shows the EDS depicting the materials detected in its chemical composition. As expected, N and Si appeared to be present in large quantities. The size of the nanoparticles was also verified in the images taken of the nanoparticles.

### 2.4. Mechanical Characterization

Several tests were conducted on the samples to examine their mechanical characteristics, including UTS, flexural and Charpy impact strength, Young’s and flexural modulus, and M-H. For the measurement of each of them, a proper apparatus was used according to the respective international standards. In particular,

- Tensile tests were conducted on V-type examples with a thickness of 3.2 mm, ASTM D638-02a [60], by placing them between the two uniform grips of the Imada MX2 apparatus, from the company Imada Inc., located in Northbrook, IL, USA.
- A flexural test (3-point bending), ASTM D790-10 [64], by the same apparatus as above, set in flexure operation, and with a 52.0 mm clearance between the supports.
- An impact test, ASTM D6110-04 [65], employing an impact (Charpy) apparatus model called MT 220, from Terco, Kungens Kurva, Sweden.
- A M-H test, ASTM E384-17 [66], by the name of Test 300, from Innova Europe BV, Maastricht, NL, USA (Vickers device), on specimens being fully polished, with 100 gF force, and 10 s of indentation duration.

### 2.5. Raman Spectra

A LabRAM HR Raman Spectrometer model produced by the company HORIBA Scientific in Kyoto, Japan, was selected for acquiring the Raman spectra. The excitation was supported by a solid-state laser module (532 nm), and the output power (maximum) was 90 mW. About 2 cm^−1^ was the Raman spectral resolution with 600 grooves grating. An LMPlanFL N Olympus (Tokyo, Japan) objective lens, with 0.5 numerical aperture, sends light to the sample and gathers the Raman signals. An objective lens magnifying 50× was operated with a clearance of 10.6 mm. The laser power limit was assigned to a neutral density filter (5% transmittance), calculated at around 4.5 mW for the sample. The volume of the measurement was 1.7 μm vertically and 2 μm longitudinally. The Raman spectral amplitude was in the range of 40 and 3900 cm^−1^, with the assistance of three optical windows. The measurement points were exposed for 10 s, and five accumulations were recorded. Visual inspection of the irradiated areas was performed, excluding discoloration or degradation due to laser irradiation.

LabSpec software v. 6 from HORIBA (Kyoto, Japan) was used for raw data processing. For each spectrum, the following procedure was used: (a) removal of cosmic rays; (b) denoising of the signal with a 5 points kernel; (c) removal of background with a 6th-grade polynomial; and (d) normalization of the spectral utilizing the maximum peak.

### 2.6. Thermal, Rheometric, and μ-CT Scan Investigation

The nanocomposites were characterized for their thermal, rheometric, and quality (voids in the 3D printing structure and geometrical accuracy) characteristics. The apparatus employed in each test is presented below, while the analytical methodology is provided in the Supplementary Materials of the study:

- TGA: Diamond Perkin Elmer (Waltham, MA, USA).
- DSC: DSC 25 calorimeter (TA Instruments, New Castle, DE, USA).
- Rheology: DHR-20 (TA Instruments, DE, USA).
- μ-CT scanning: Tomoscope HV Compact 225 kV Micro Focus CT-scanner (Werth Messtechnik GmbH, Giessen, Germany).

### 2.7. Quality Control

In this research, quality control was applied in all of the steps of the process. The most critical steps are highlighted in the manuscript. More specifically, initially, the produced filament underwent quality control. This is a critical step since good quality filament contributes to the production of better-quality 3D-printed parts and fewer processability issues. The quality of the filament was evaluated through the real-time measurement of its diameter throughout its production process, and manual sample measurements of its diameter at randomly selected positions after its production. It was found that the produced filament diameter was maintained within an acceptable range for MEX 3D printing. The protocol in this case was to produce filament with a diameter in the range of 1.65–1.85 mm. Apart from the diameter measurements, its side surface was inspected on the microscope for defects, voids, and discontinuities in the morphology. This was a qualitative method with no specific quantitative metrics.

Afterward, the 3D-printed samples were also quality tested. Initially, their dimensions were measured manually with a high-quality caliper. The samples’ side surface was inspected with SEM to evaluate the uniformity of the layers, the fusion between them, and the existence of defects or voids. Same as before, this was a qualitative method with no specific quantitative metrics. Finally, two quality metrics were evaluated in this research with sophisticated computer tomography scanning, which provides accuracy and reliability in the measurements. These were the dimensional deviation in the 3D-printed samples and the porosity in their structure. This process provided quantitative measures, which are presented in the study, but no specific quality control protocol regarding the acceptable values range was defined. This was because it is common for the 3D-printed parts to vary in their dimensional accuracy and voids in the 3D printing structure.

## 3. Results

### 3.1. Raman Outcome

**Figure 3:**
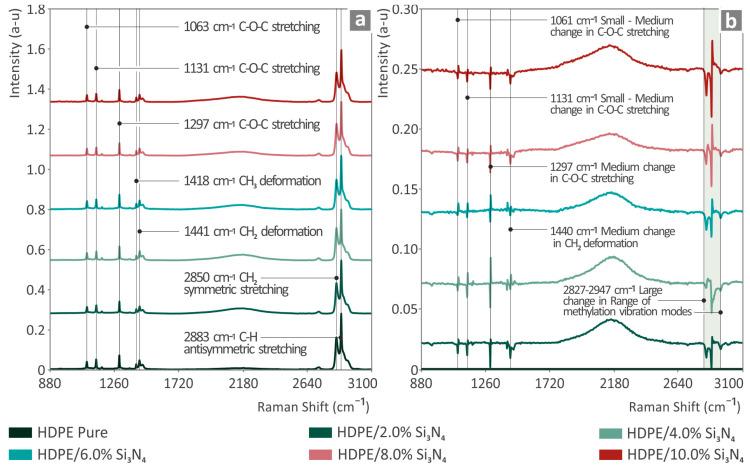
(**a**) Raman spectra from pure HDPE, HDPE/Si_3_N_4_ 2 wt. %, 4 wt. %, 6 wt. %, 8 wt. %, 10 wt. %; (**b**) Raman spectral HDPE/Si_3_N_4_ 2 wt. %, HDPE/Si_3_N_4_ 4 wt. %, HDPE/Si_3_N_4_ 6 wt. %, HDPE/Si_3_N_4_ 8 wt. %, and HDPE/Si_3_N_4_ 10 wt. % from HDPE/pure differences.

In Figure 3a, the Raman peaks for the unfilled HDPE item are labeled. These are also compiled in Table S1 of the Supplementary Materials in comparison with the bibliography [67,68,69,70,71].

In Figure 3a, it can be observed that the Raman peaks identified from the total amount of the samples are from the unfilled HDPE. CH_3_ and CH_2_ deformations were found at 1418 and 1441 cm^−1^. C-O-C stretching was detected at 1063, 1131, and 1297 cm^−1^. CH_2_ symmetric stretching was located at 2850 cm^−1^, and C-H antisymmetric stretching was located at 2883 cm^−1^. As shown in Figure 3b, as the content of Si_3_N_4_ is raised in HDPE, the respective Raman lines of unfilled HDPE are differentiated in intensity. The broad photoluminescence seems to expand with an increasing Si_3_N_4_ percentage between 1800–2600 cm^−1^.

The introduction of Si_3_N_4_ in HDPE lead to a reduction in the Raman lines at 1061 cm^−1^ (C-O-C stretching [67]), 1131 cm^−1^ (C-C symmetric vibration [69]), 1297 cm^−1^ (skeletal stretching, C-O-C bonds [69,70]), 1440 cm^−1^ (C-H_3_ deformation [67,69]; C-H_2_ deformation [67,69]; C-H_3_ symmetric bending [67,68,72]), and 2854 cm^−1^ (C-H_2_ symmetric stretching [70]). All of the aforementioned data are presented in Table 1.

### 3.2. TGA and DSC Findings

**Figure 4:**
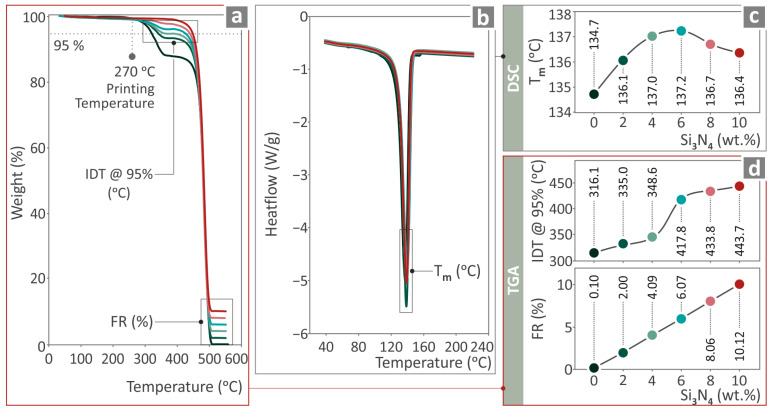
(**a**) Weight vs. temperature graphs of HDPE/Si_3_N_4_ 0.0, 2.0, 4.0, 6.0, 8.0, and 10.0 wt. %; (**b**) heat-flow vs. temperature graphs of HDPE/Si_3_N_4_ 0.0, 2.0, 4.0, 6.0, 8.0, and 10.0 wt. %; (**c**) Tm, (**d**) FR, and IDT values.

Figure 4 presents the TGA and DSC results for the HDPE/Si_3_N_4_ samples, in TGA and DSC graphs. In Figure 4a, the weight for the temperature graph (TGA) of HDPE/Si_3_N_4_ is 0.0, 2.0, 4.0, 6.0, 8.0, and 10.0 wt. % is shown, while the measured final residue (FR) and initial decomposition temperature (IDT) values are shown in Figure 4d. Figure 4b illustrates the heat flow compared to the temperature graph (DSC) of HDPE/Si_3_N_4_ 0.0, 2.0, 4.0, 6.0, 8.0, and 10.0 wt. % samples, while in Figure 4c there are the T_m_ values. As shown, the addition of Si_3_N_4_ in the HDPE increases the stability of the matrix by shifting the beginning of the degradation of the material to higher temperatures. The residual mass agrees with the content of the Si_3_N_4_ nanoparticles in the nanocomposites, as the Si_3_N_4_ is not expected to degrade at such temperatures. Additionally, the 3D printing temperature is not close to the temperature ranges of the neat HDPE, and the nanocomposites start to degrade. Regarding the DSC results, the insertion of Si_3_N_4_ nanoparticles in the HDPE thermoplastic increases the melt temperature of HDPE, with the higher value reported for the 6 wt. % content nanocomposite. Then, the Tm starts to decrease, but it is still higher than the neat HDPE.

### 3.3. Rheology Results

**Figure 5:**
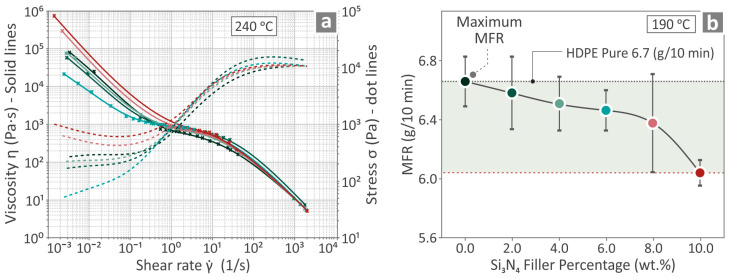
Unfilled HDPE and HDPE/Si_3_N_4_ 2.0, 4.0, 6.0, 8.0, and 10.0 wt. % results in (**a**) viscosity graphs and (**b**) MFR. Different colors refer to different filler content in the nanocomposites.

Figure 5a illustrates the viscosity stress compared to the shear rate graphs for HDPE/Si_3_N_4_ 0.0, 2.0, 4.0, 6.0, 8.0, and 10.0 wt. %, at 240 °C. As the viscosity levels (solid lines) decreased, the stress levels (dotted lines) increased. In Figure 5b, the MFR results of HDPE/Si_3_N_4_ samples at 190 °C are depicted. As can be seen, the MFR levels were lowered when the Si_3_N_4_ nanoparticle content was raised.

### 3.4. Filament Inspection and Testing Results

**Figure 6:**
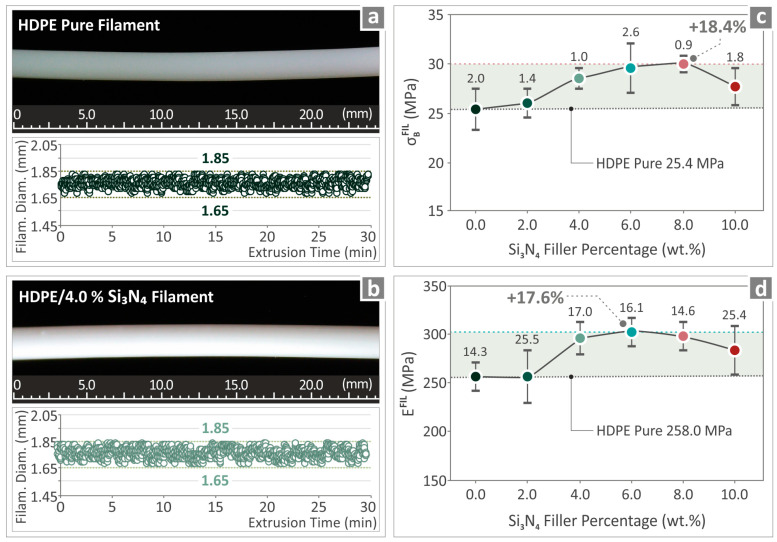
(**a**) Unfilled HDPE filament image and results from diameter monitoring, (**b**) HDPE/Si_3_N_4_ 4.0 wt. % filament image and the diameter captured measurements, (**c**) UTS levels, and (**d**) Young’s modulus levels from the testing of the HDPE/Si_3_N_4_ 0.0, 2.0, 4.0, 6.0, 8.0, and 10.0 wt. % filaments.

Figure 6a,b show two images captured from a randomly chosen section of the filaments made of neat and HDPE/Si_3_N_4_ 4.0 wt. %, and the filament diameter monitoring results. Both filaments were of excellent quality, and their diameter remained steady, ranging from 1.65 to 1.85 mm. Figure 6c,d illustrate the experimental findings of the tensile strength and Young’s modulus of all filaments.

It can be observed that, for the tensile strength, the HDPE/Si_3_N_4_ 8.0 wt. % samples presented an increase over unfilled HDPE of 18.4%. It should be noted that the addition of Si_3_N_4_ had a positive influence on all composites with different filler percentages. Considering the tensile modulus of elasticity, the HDPE/Si_3_N_4_ 6.0 wt. % was measured to be 17.6% higher than the levels of unfilled HDPE.

### 3.5. Mechanical Tests

**Figure 7:**
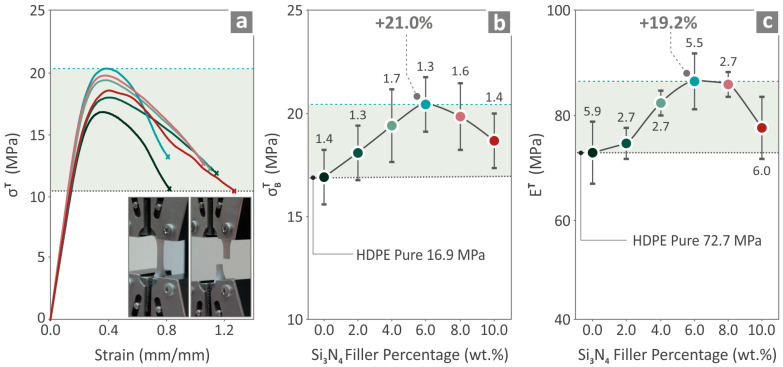
HDPE/Si_3_N_4_ 0.0–10.0 wt. % measured tensile values, (**a**) stress compared to strain curves, (**b**) UTS, and (**c**) modulus of elasticity. Different colors refer to different filler content in the nanocomposites.

**Figure 8:**
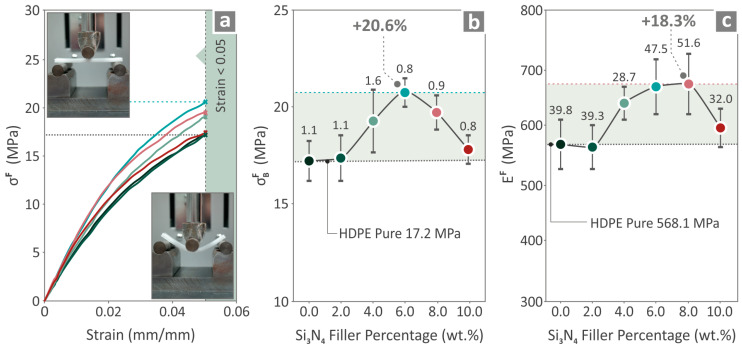
HDPE/Si_3_N_4_ 0.0–10.0 wt. % measured values for flexural characteristics, (**a**) stress compared to strain curves, (**b**) flexural strength, and (**c**) modulus. Different colors refer to different filler content in the nanocomposites.

**Figure 9:**
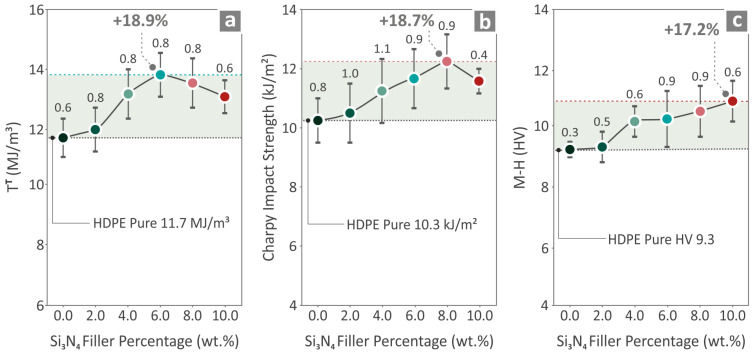
HDPE/Si_3_N_4_ 0.0–10.0 wt. % measured values for (**a**) tensile toughness, (**b**) impact (Charpy) strength, and (**c**) M-H.

In Figure 7, Figure 8 and Figure 9, the values measured for the HDPE/Si_3_N_4_ are 0.0–10.0 wt. % (2.0 step) specimens’ mechanical characteristics are depicted. Figure 7a illustrates the tensile stress-strain results, also presenting two pictures from the experimental procedure of a randomly selected tensile sample. Figure 7b,c depict the UTS and Young’s modulus results and highlight that the HDPE/Si_3_N_4_ 6.0 wt. % composite revealed the most remarkable results in relation to the unfilled HDPE, with an increase in the tensile strength and Young’s modulus by 21.0% and 19.2%, respectively. It should be noted that all of the composite values were improved compared to the unfilled HDPE.

Figure 8a shows the flexural stress compared to the strain results, and two captures from the experimental procedure of a flexural specimen. Figure 8b presents the flexural strength, revealing that the HDPE/Si_3_N_4_ 6.0 wt. % composite had the greatest levels compared to the unfilled HDPE, by 20.6%. On the other hand, the information provided in Figure 8c regarding the flexural modulus of elasticity shows that HDPE/Si_3_N_4_ of 4.0 wt. % filler quantity was the most positively affected, compared to the unfilled HDPE, by 18.3%.

Figure 9a provides information about the tensile toughness, which shows that the composite that possessed the most increased results compared to pure HDPE was the HDPE/Si_3_N_4_ 6.0 wt. % (18.9%). Figure 9b illustrates the Charpy impact strength findings and reveals that HDPE/Si_3_N_4_ 8.0 wt. % had an 18.7% higher impact strength than the neat HDPE. Figure 9c presents the M-H outcome, where the composite of HDPE/Si_3_N_4_ 10.0 wt. % appears to have the highest levels, 17.2% higher than the unfilled HDPE. In the Supplementary Materials of this work, there is a summarization of some of the examined characteristics of all of the HDPE/Si_3_N_4_ composite samples (Figure S2). In particular, the tensile and flexural strength results, as well as the A2N@95% dimensional deviation and porosity (presented in detail below) of the specimens, are shown in four different spider-shaped graphs.

### 3.6. Specimens’ μ-CT Scan Characterization

**Figure 10:**
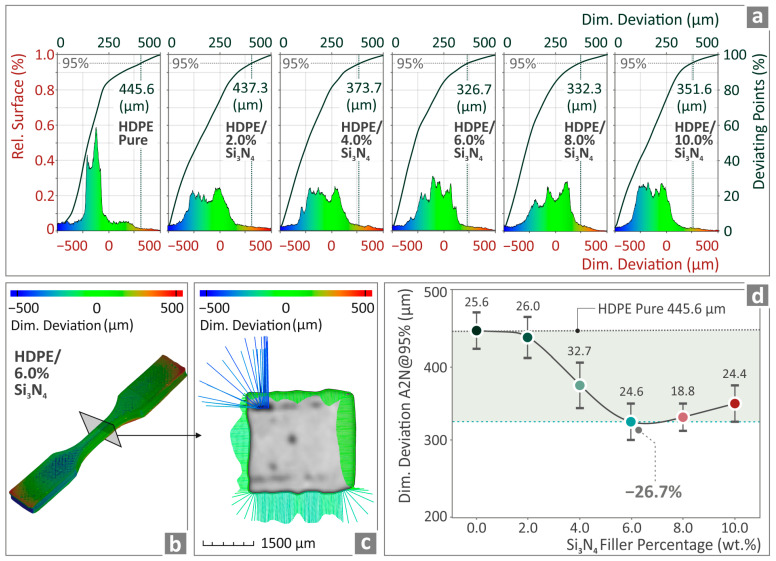
(**a**) Unfilled HDPE and HDPE/Si_3_N_4_ 2.0–10.0 wt. % dimensional deviation curves, (**b**,**c**) HDPE/Si_3_N_4_ 6.0 wt. % tensile sample by color coding, (**d**) geometry deviation levels of HDPE/Si_3_N_4_ 0.0–10.0 wt. %.

**Figure 11:**
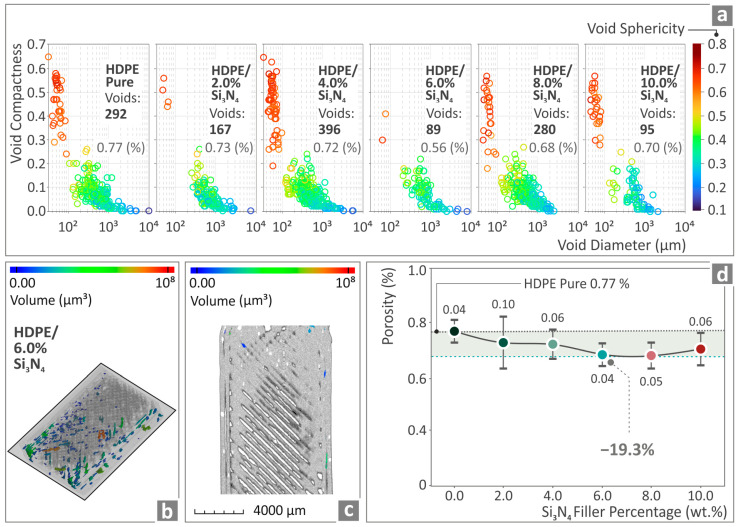
(**a**) HDPE/Si_3_N_4_ 2.0–10.0 wt. % and unfilled HDPE porosity results, (**b**,**c**) porosity of HDPE/Si_3_N_4_ 6.0 wt. % example through color coding, (**d**) porosity levels of HDPE/Si_3_N_4_ 0.0–10.0 wt. %.

Figure 10 and Figure 11 show the outcomes derived from the conduction of the micro-CT scan, with regard to the measured dimensional deviation and porosity of the samples. Figure 10a shows graphs of the related surface versus the dimensional deviation of HDPE/Si_3_N_4_ 0.0–10.0 wt. %. In Figure 10b,c, color coding is used for the dimensional deviation projection belonging to the HDPE/Si_3_N_4_ 6.0 wt. % composite tensile example. In Figure 10d, the levels of dimensional deviation with respect to the Si_3_N_4_ nanoparticles percentage are presented for all of the HDPE/Si_3_N_4_ composites. HDPE/Si_3_N_4_ 6.0 wt. % seems to be the composite with the greatest improvement, with a 26.7% reduction in relation to the unfilled HDPE (more accurate geometry).

In Figure 11a, diagrams of sphericity and void compactness versus the diameter of HDPE/Si_3_N_4_ nanocomposites are presented. As illustrated in Figure 11b,c, color coding was employed to show the voids of the HDPE/Si_3_N_4_ 6.0 wt. % composite sample. Figure 11d depicts the porosity levels versus the Si_3_N_4_ filler quantity of all HDPE/Si_3_N_4_ composites shown. The HDPE/Si_3_N_4_ 6.0 wt. % levels appear to have the most positively influenced performance, being 19.3% lower than the unfilled HDPE (lesser porosity percentage).

### 3.7. Morphological Results of the Specimens

**Figure 12:**
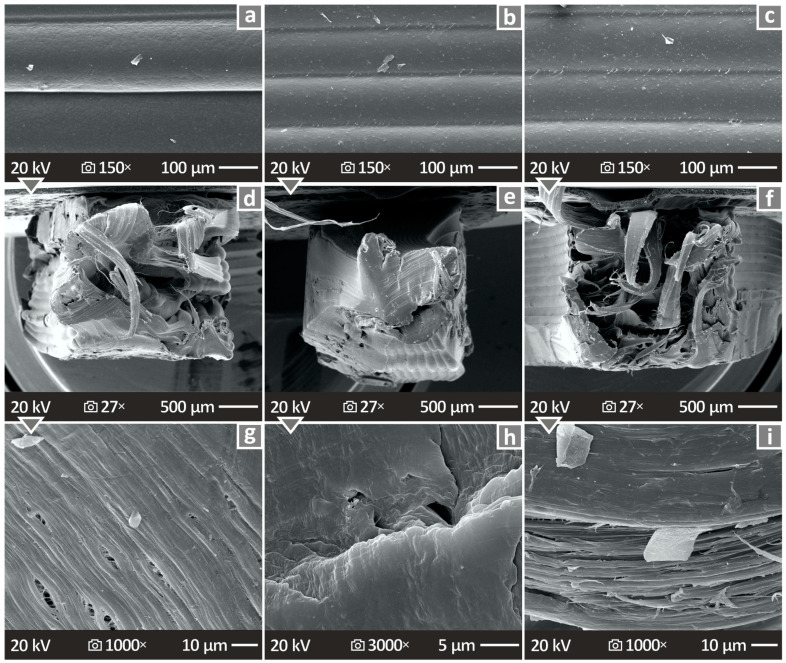
Unfilled HDPE, HDPE/Si_3_N_4_ 4.0 wt. % along with the 8.0 wt. % content nanocomposite SEM pictures capturing (**a**–**c**) the lateral surfaces in 150× magnification, (**d**–**f**) the fracture section in 27×, and (**g**–**i**) 1000× magnifications.

By inspecting the images provided in Figure 12, the lateral surfaces appear to have good layering without voids and important defects. On the other hand, the fracture surfaces present obvious ductility at lower magnifications, while the higher magnifications show the existence of pores and voids.

**Figure 13:**
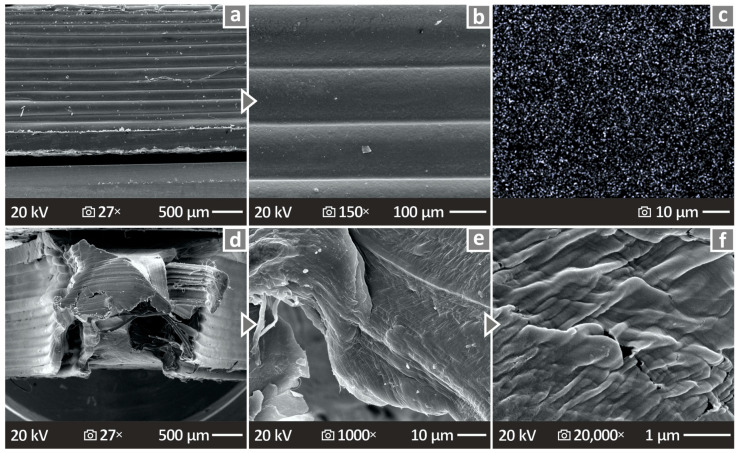
(**a**–**c**) HDPE/Si_3_N_4_ 10.0 wt. % SEM images of the vertical surface in 27× magnification, (**d**) EDS mapping presenting the material dispersion (Si), and (**e**,**f**) HDPE/Si_3_N_4_ 10.0 wt. % SEM pictures of the fracture section in 1000× magnification.

Figure 13 shows the SEM results for the HDPE/Si_3_N_4_ 10.0 wt. % composite, as well as the results from the conduction of EDS. Figure 13a,b show images from the lateral surface at two different magnifications, 27× and 150×, revealing significant layering. Figure 13c shows the EDS mapping image, indicating material dispersion, which appears to be very well distributed. Therefore, even for the higher-loaded nanocomposites, no particle clustering was located, showing that the method followed for the nanocomposites’ preparation was effective in terms of the particles’ dispersion in the polymeric matrix. Figure 13d–f show the fracture cross-section images at 27×, 1K×, and 20K× magnification. In these cases, the samples were again characterized by ductility, but there was not much porosity.

## 4. Discussion

The tests carried out herein provided useful information regarding the impact of the Si_3_N_4_ additive (nanoparticles) on the matrix material of the HDPE thermoplastic. Several tests were conducted to characterize the behavior of the samples. These were related to thermal characterization, rheology, mechanical behavior, structure, and morphology. The thermal investigation included TGA and DSC, which indicated that the addition of the Si_3_N_4_ filler caused an increase in the FR, IDT, and T_m_ values. It should be noted that no statistical analysis was carried out in the study. This was because all of the different filler content composites were prepared and tested with the same settings to have comparable results. The only parameter that changed was the percentage of the filler in the compound. The study aimed to evaluate the effect of the Si_3_N_4_ ceramic on the HDPE thermoplastic when composites are prepared with the material extrusion method. Studying the effect of different parameter values was not within the scope of the study; therefore, there was no practical merit in conducting a statistical analysis in this case.

As for the rheological investigation, the MFR levels decreased with the addition of Si_3_N_4_ to the HDPE. This finding, along with the thermal measurements, which indicated an increased Tm with the increase in the nanoparticle content for the samples, suggests that the 3D printing settings need to be rescheduled to achieve maximum performance. Then, common settings optimized for the unfilled HDPE were applied from the literature to all nanocomposites to be able to correlate the experimental results.

The fabricated filaments underwent inspection and mechanical testing, where it was pointed out that the HDPE/Si_3_N_4_ 8.0 wt. % composite sample was the one with the most improved tensile strength performance (18.4%). Then, the HDPE/Si_3_N_4_ 6.0 wt. % presented the most remarkable levels of tensile modulus of elasticity (17.6%).

Among the 3D-printed samples, the composite that presented the most improved results was the HDPE/Si_3_N_4_ 6.0 wt. % for four mechanical properties out of the seven. The UTS and Young’s modulus were improved by 21.0% and 19.2%, respectively, while the flexural strength and tensile toughness were improved by 20.6% and 18.9%, respectively, compared to those of the neat HDPE. Regarding the variation in tensile strength among different samples, the calculated variation is within acceptable limits for 3D-printed parts. Three-dimensional printed parts usually show higher deviation in their mechanical properties than bulk parts due to their 3D printing structure, which affects the uniformity in the parts and induces anisotropy. This is a known and common issue when testing 3D-printed parts [73,74].

Then HDPE/Si_3_N_4_ 8.0 wt. % also presented great improvement compared to pure HDPE for flexural modulus by 18.3% and Charpy impact strength by 18.7. Finally, the highest microhardness was found to be in the nanocomposite having Si_3_N_4_ 10.0 wt. % content, by 17.2%. This is expected, as ceramics are popular for their hardness and wear-resistance performances. Therefore, high filler-content nanocomposites were found to have better M-H measurements.

Microcomputed tomography-derived information also presented a positively influenced behavior. The geometrical inaccuracy and voids in the structure (porosity) of the specimens were reduced by 26.7% and 19.3%, respectively, in the case of HDPE/Si_3_N_4_ 6.0 wt. %. This shows that good quality metrics lead to better mechanical performance as well. The reduced porosity in the 3D printing was already expected to improve the mechanical performance, as already stated in the literature [75,76].

With regard to the SEM results, the images reveal important information regarding the morphology of the various fabricated specimens. The pictures possessed herein indicated a very good distribution of the layering without defects by observing the lateral surface. The fractured surfaces, which were also examined, indicated very ductile behavior, as well as the presence of voids and porosity. SEM and EDS were also used to evaluate the nanoparticle dispersion in the HDPE polymeric matrix. As mentioned earlier, no particle clustering was observed in the SEM captures or the EDS mapping for the Si element. At the same time, the mechanical test deviation was not elevated, indicating a similar composition between the parts in each case. This can lead to the assumption that the methodology developed for the nanocomposites’ preparation achieved good nanoparticle dispersion in the HDPE thermoplastic, even at the highest loading tested. Regarding the highest filler loading investigated, the mechanical tests showed a constant reduction in the mechanical response of the parts at filler contents higher than 6 wt. %, which indicated saturation of the Si_3_N_4_ nanoparticles in the HDPE thermoplastic. Therefore, there was no reason to test even higher loadings than the ones tested. The saturation threshold estimation was not within the content of the research.

As the bibliography search revealed, no similar nanocomposites have been tested for their mechanical performance in 3D printing with the MEX method. To assess the outcome of the research with the literature, the effect of different additives on the HDPE matrix was compared to the findings of the current research (Table 2). As shown, the reinforcing performance of Si_3_N_4_ to the HDPE matrix was similar to that of titanium nitride [33], while the other presented additives achieved better reinforcement results. Still, it should be mentioned that each filler is appropriate for specific applications, due to their overall characteristics. Carbon black [63] achieved the highest reinforcement among the presented additives. Still, this was achieved at a high loading of 20 wt. %, which was not possible to be achieved herein. Interestingly, the 6 wt. % filler content was the one achieving the better results in four out of the six fillers presented; it should be noted that they are different in nature and refer to diverse applications.

Furthermore, the reinforcing effect of Si_3_N_4_ nanoparticles on different types of matrix materials in AM was compared and presented in Table 3. As shown, with the exception of PLA [56], the reinforcing effect of the Si_3_N_4_ nanoparticles is rather similar between the different polymeric materials. This is a strong indication of the reliability of the presented findings in this research. The highest reinforcing effect on PLA [56] can be owed to varied matrix–additive interactions. The comparisons presented in the two Tables reveal the limitations of the study. As the presented results differ, this instructs that the current findings cannot be generalized to other polymers or fillers. Additionally, the preparation method affects the results. Therefore, individual research endeavors are required in each method and matrix–filler combination.

## 5. Conclusions

The combination of the HDPE matrix material with Si_3_N_4_ was the main objective of this research, to enhance the mechanical performance within the context of MEX 3D printing. Samples were used to investigate their mechanical behavior and thermal, rheological, structural, and morphological characteristics by performing a variety of tests and analyses. The main findings were as follows:

- The 6.0 wt. % composite reinforced the matrix in most of the mechanical characteristics (more than 20% higher tensile and flexural strength compared to the unfilled HDPE).
- The structural characteristics were improved.
- Quality characteristics related to the porosity of the internal structure and the dimensional accuracy were improved with the introduction of the Si_3_N_4_ filler in the HDPE polymer.
- A thermomechanical method was followed, which can be easily scaled up for industrial use.

These findings can lead to better 3D-printed objects with the MEX method made of the HDPE polymer, having a more robust performance. Future work could be devoted to the examination of composites with different filler percentages. The 3D printing parameters can be further optimized for the 6 wt. % nanocomposite, achieving even better enhancement in the mechanical response. Finally, the same settings were used in all cases for comparison purposes.

## Figures and Tables

**Table 1:** Raman peak significant differences of HDPE/Si3N4 examples from unfilled HDPE.

1061	Peak drop	Small-medium change in C-O-C stretching [67]
1131	Peak drop	Small-medium change in C-O-C stretching [68]
1297	Peak drop	Medium change in C-O-C stretching [67]
1440	Peak drop	Medium change in CH_2_ deformation [67,69]
2827–2947	Inconsistent behavior	Large change in the range of methylation vibration modes [70]

**Table 2:** Various additives reinforcing efficacy on HDPE in MEX 3D printing.

Increase (%)	Si_3_N_4_	Biochar [30]	Carbon Black [63]	Copper [31]	Titanium Dioxide [32]	Titanium Nitride [33]
Tensile strength	21.0	37.8	57.8	31.2	28.5	24.3
Flex. strength	20.6	35.9	59.7	36.7	77.6	28.7
Opt. loading	6.0	6.0	20.0	6.0	10.0, 2.5	6.0

**Table 3:** Reinforcing effect of Si_3_N_4_ on various polymeric matrices in AM.

Increase (%)	HDPE	(PP) [57]	PLA [56]	(PETG) [55]	ABS [58]	Biomed Resin [59]
Tensile strength	21.0	16.0	40.4	24.5	25.6	23.6
Flex. strength	20.6	15.7	68.0	16.6	29.4	44.8
Opt. loading	6.0	2.0	6.0	6.0	6.0	1.0

## Data Availability

The raw/processed data required to reproduce these findings cannot be shared because of technical or time limitations.

## Supplementary Materials

The following supporting information can be downloaded at: https://www.mdpi.com/article/10.3390/nano14201680/s1, Figure S1: Applied settings for the 3D-P of the specimens, pictures from the tensile, flexural, and impact 3D-P specimen samples, as well as the designed models of those specimens with their dimensions and respective ASTM international standards. Table S1: Significant Raman peaks and their corresponding assignments for pure HDPE. Figure S2: The results from all the HDPE/Si_3_N_4_ are 0.0, 2.0, 4,0, 6.0, 8.0, and 10.0% tested samples regarding their (a) tensile strength, (b) flexural strength, (c) A2N@95% and (d) voids percentage, gathered in four spider-shaped graphs.
