# Supplementary material for: Printability Metrics and Engineering Response of HDPE/Si3N4 Nanocomposites in MEX Additive Manufacturing
Source: Nanomaterials (Basel). 2024 Oct 19;14(20):1680. doi: 10.3390/nano14201680 (PMC11510129; doi:10.3390/nano14201680)
Supplement: Supplementary file 1 [file nanomaterials-14-01680-s001.zip › nanomaterials-3250626-supplementary.pdf]

nanomaterials

Article

Printability Metrics and Engineering Response of HDPE/Si_3N_4 Nanocomposites in MEX Additive Manufacturing

Vassilis Papadakis [1,2], Markos Petousis [3], Nikolaos Michailidis [4,5], Mariza Spiridaki [3], Ioannis Valsamos [3], Apostolos Argyros [4,5], Katerina Gkagkanatsiou [3], Amalia Moutsopoulou [3], Nectarios Vidakis [3*]

1 Department of Industrial Design and Production Engineering, University of West Attica, 122 43 Athens, Greece, v.papadakis@uniwa.gr;
2 Foundation for Research and Technology Hellas (FORTH), Institute of Electronic Structure and Laser (IESL), 70013, Heraklion, Greece
3 Department of Mechanical Engineering, Hellenic Mediterranean University, Heraklion 71410, Greece, markospetousis@hmu.gr; mspyridaki@hmu.gr; valsamos@hmu.gr; gkagka@hmu.gr; amalia@hmu.gr; vidakis@hmu.gr;
4 Physical Metallurgy Laboratory, Mechanical Engineering Department, School of Engineering, Aristotle University of Thessaloniki, 54124 Thessaloniki, Greece, nmichail@auth.gr; aargyros@auth.gr
5 Centre for Research & Development of Advanced Materials (CERDAM), Center for Interdisciplinary Research and Innovation, Balkan Centre, Building B', 10th km Thessaloniki-Thermi road, 57001, Thessaloniki, Greece
* Correspondence: e-mail: vidakis@hmu.gr (Nectarios Vidakis), Tel.: +302810379227

S1. 3D printing settings, geometry of the samples per test and infill structure

Parameters	Value	Units
HDPE Si_3N_4 content	0.0–2.0–4.0–6.0–8.0–10.0	%
Printing orientation	±45	deg
Nozzle temperature	230	°C
Bed temperature	90	°C
Layer thickness	0.2	mm
Number of perimeters	2	–
Fill density	100	%
Travel speed	40	mm/sec

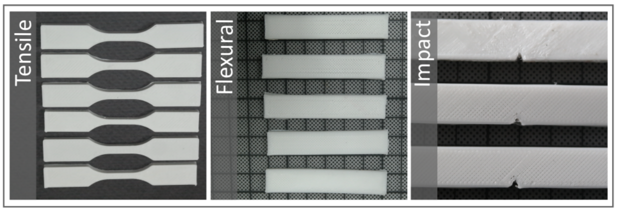

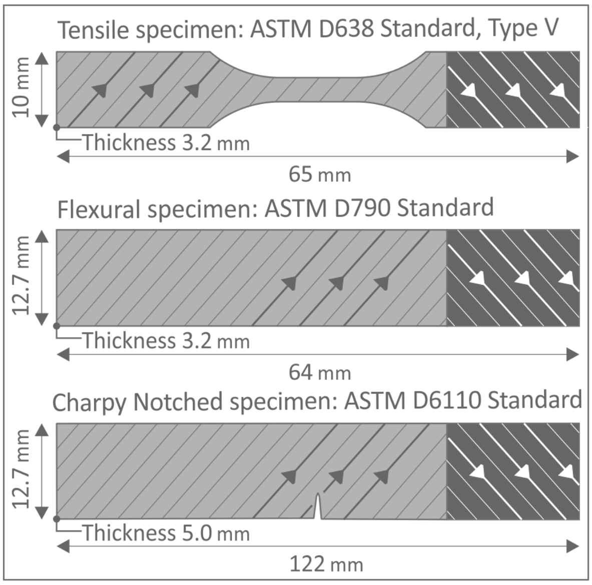

Figure. S1. Applied settings for the 3D-P of the specimens, pictures from the tensile, flexural and impact 3D-P specimen samples, as well as the designed models of those specimens with their dimensions and respective ASTM international standards.

S2. Thermal investigation

TGA and DSC were used to assess the thermal characteristics of HDPE/Si_3N_4 samples. A Diamond Perkin Elmer (Massachusetts, USA) device was selected for the TGA with a temperature cycle of 40–550 °C and a rate of increase of 10 °C / min. A Discovery Series DSC-25 DSC calorimeter (TA Instruments, Delaware, United States) was used to conduct the DSC, with an RSC-90 Refrigerated Cooling System. The working conditions during the TGA and DSC analyses were an inert environment of high-purity N_2 (nitrogen gas).

S3. Rheometric analysis

A DHR-20 Discovery Hybrid Rotational Rheometer (TA Instruments, Delaware, USA) was employed for the rheometric investigation (ASTM D1238-13, for MFR). An Environmental Test Chamber (parallel-plate setup for temperature regulation) was used in the rheometric analysis. The acquisition duration was 10 seconds for the measured points to prevent excessive heating and decomposition. Both MFR and rotational rheometric tests were conducted to assess the flow rates of the materials at selected temperatures and preselected pressures.

S4. μ-CT scan

The porosity percentage and dimensional deviations of the 3D fabricated samples were evaluated through microcomputed tomography (μ-CT) scans. A Tomoscope HV Compact 225 kV Micro Focus CT-scanner from Werth Messtechnik GmbH located in Giessen, Germany, accompanied by a 1024 × 1024-pixel sensor was used, alongside a software named VG Studio MAX 2.2 from Volume Graphics GmbH, Heidelberg, Germany, for data analysis. A 75 L setup with a resolution of 72.58 μm on the X-axis and 72.65 μm on the Y-axis was used for the dimensional accuracy measuring. A 16 L setup with resolution 15.46 μm on the X-axis and 15.49 μm on the Y-axis, was used for the porosity measuring. Both included 1600 sections/ revolution.

S5. Raman peaks

Table S1. Significant Raman peaks and their corresponding assignments for pure HDPE.

Wavenumber (cm^{-1})	Intensity	Raman peak assignment
1063	Medium	C-O-C stretching [1]
1131	Medium	C-O-C stretching [2]
1297	Medium	C-O-C stretching [1]
1418	Small	CH_3 deformation [1]
1441	Small	CH_2 deformation [1,3]
2850	Major	CH_2 symmetric stretching [4]
2883	Major	C-H antisymmetric stretching [5]

S6. Summary of the main experimental findings

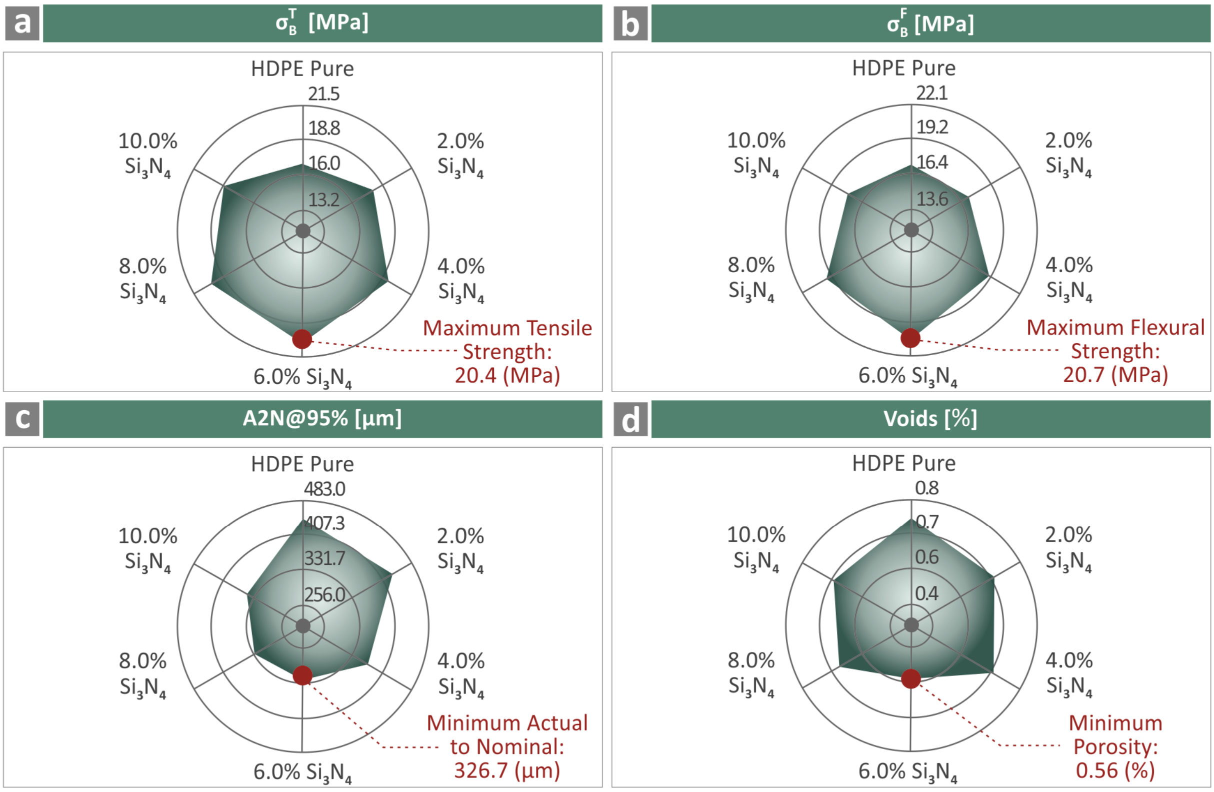

Figure S2. The results from all the HDPE/ Si3N4 0.0, 2.0, 4,0. 6.0, 8.0 and 10.0% tested samples regarding their **(a)** tensile strength, **(b)** flexural strength, **(c)** A2N@95% and **(d)** voids percentage, gathered in four spider-shaped graphs.

References

1. Stuart, B.H. Temperature Studies of Polycarbonate Using Fourier Transform Raman Spectroscopy. *Polymer Bulletin* **1996**, *36*, 341–346, doi:10.1007/BF00319235.
2. Resta, V.; Quarta, G.; Lomascolo, M.; Maruccio, L.; Calcagnile, L. Raman and Photoluminescence Spectroscopy of Polycarbonate Matrices Irradiated with Different Energy 28Si+ Ions. *Vacuum* **2015**, *116*, 82–89, doi:10.1016/j.vacuum.2015.03.005.
3. Zimmerer, C.; Matulaitiene, I.; Niaura, G.; Reuter, U.; Janke, A.; Boldt, R.; Sablinskas, V.; Steiner, G. Nondestructive Characterization of the Polycarbonate - Octadecylamine Interface by Surface Enhanced Raman Spectroscopy. *Polym Test* **2019**, *73*, 152–158, doi:10.1016/j.polymertesting.2018.11.023.
4. Makarem, M.; Lee, C.M.; Kafle, K.; Huang, S.; Chae, I.; Yang, H.; Kubicki, J.D.; Kim, S.H. Probing Cellulose Structures with Vibrational Spectroscopy. *Cellulose* **2019**, *26*, 35–79, doi:10.1007/s10570-018-2199-z.
5. Liu, X.; Zou, Y.; Li, W.; Cao, G.; Chen, W. Kinetics of Thermo-Oxidative and Thermal Degradation of Poly(d,l-Lactide) (PDLLA) at Processing Temperature. *Polym Degrad Stab* **2006**, *91*, 3259–3265, doi:10.1016/j.polymdegradstab.2006.07.004.

Disclaimer/Publisher's Note: The statements, opinions and data contained in all publications are solely those of the individual author(s) and contributor(s) and not of MDPI and/or the editor(s). MDPI and/or the editor(s) disclaim responsibility for any injury to people or property resulting from any ideas, methods, instructions or products referred to in the content.
